# LLIN Evaluation in Uganda Project (LLINEUP2)—Factors associated with coverage and use of long‑lasting insecticidal nets following the 2020–21 national mass distribution campaign: a cross-sectional survey of 12 districts

**DOI:** 10.1186/s12936-022-04302-7

**Published:** 2022-10-19

**Authors:** Jaffer Okiring, Samuel Gonahasa, Martha Nassali, Jane F. Namuganga, Irene Bagala, Catherine Maiteki‑Sebuguzi, Jimmy Opigo, Isaiah Nabende, Joanita Nangendo, Jane Kabami, Isaac Ssewanyana, Steven M. Kiwuwa, Joaniter I. Nankabirwa, Grant Dorsey, Jessica Briggs, Moses R. Kamya, Sarah G. Staedke

**Affiliations:** 1 Clinical Epidemiology Unit, School of Medicine, Makerere University College of Health Sciences, Kampala, Uganda; 2 Infectious Diseases Research Collaboration, PO Box 7475, Kampala, Uganda; 3 National Malaria Control Division, Ministry of Health, Kampala, Uganda; 4 Department of Child Health and Development Centre, School of Medicine, Makerere University College of Health Sciences, Kampala, Uganda; 5 Department of Medicine, University of California San Francisco, San Francisco, USA; 6 Department of Medicine, Makerere University, Kampala, Uganda; 7 Department of Clinical Research, London School of Hygiene & Tropical Medicine, London, UK

**Keywords:** Malaria, Long-lasting insecticidal nets (LLINs), Uganda, Mass distribution campaign, Ownership, Coverage

## Abstract

**Background:**

In 2020–2021, long-lasting insecticidal nets (LLINs) were distributed nationwide in Uganda during the COVID-19 pandemic. A cross-sectional survey was conducted in 12 districts to evaluate the impact of the campaign 1–5 months after LLIN distribution.

**Methods:**

During April–May 2021, households were randomly selected from target areas (1–7 villages) surrounding 12 government-run health facilities established as Malaria Reference Centres; at least 50 households were enrolled per cluster. Outcomes included household ownership of LLINs distributed through the universal coverage campaign (UCC) (at least one UCC LLIN), adequate coverage of UCC LLINs (at least one UCC LLIN per 2 residents), and use of LLINs (resident slept under a LLIN the previous night). Multivariate logistic regression models were used to identify household- and individual-level factors associated with outcomes, controlling for clustering around health facilities.

**Results:**

In total, 634 households, with 3342 residents and 1631 bed-nets, were included. Most households (93.4%) owned at least 1 UCC LLIN, but only 56.8% were adequately covered by UCC LLINs. In an adjusted analysis, the factor most strongly associated with adequate coverage by UCC LLINs was fewer household residents (1–4 vs 7–14; adjusted odds ratio [aOR] 12.96, 95% CI 4.76–35.26, p < 0.001; 5–6 vs 7–14 residents; aOR 2.99, 95% CI 1.21–7.42, p = 0.018). Of the 3166 residents of households that owned at least one UCC LLIN, only 1684 (53.2%) lived in adequately covered households; 89.9% of these used an LLIN the previous night, compared to 1034 (69.8%) of 1482 residents living in inadequately covered households. In an adjusted analysis, restricted to residents of inadequately covered households, LLIN use was higher in children under-five than those aged 5–15 years (aOR 3.04, 95% CI 2.08–4.46, p < 0.001), and higher in household heads than distantly-related residents (aOR 3.94, 95% CI 2.38–6.51, p < 0.001).

**Conclusions:**

Uganda’s 2021–21 campaign was successful, despite the COVID-19 pandemic. In future campaigns, strategies should be adopted to ensure high LLIN coverage, particularly for larger households. A better understanding of the drivers of LLIN use within households is needed to guide future interventions, educational messages, and behaviour change communication strategies; school-aged children and distantly-related residents appear vulnerable and could be targeted.

**Supplementary Information:**

The online version contains supplementary material available at 10.1186/s12936-022-04302-7.

## Background

Malaria remains a life-threatening public health concern, particularly in sub-Saharan Africa [1]. Long-lasting insecticidal nets (LLINs) have been shown to reduce malaria burden and mortality [2, 3], and are the primary vector control tool in Africa. Ownership of LLINs has increased markedly over the past 20 years, contributing to substantial progress in malaria control [4]. Between 2000 and 2019, an estimated 1.5 billion malaria cases were averted, 94% in Africa [1]; much of this success has been attributed to the scale-up of LLINs [5]. Recent evidence, however, suggests progress has stalled, particularly in high burden countries such as Uganda [5, 6]. To achieve universal coverage with LLINs (one LLIN for every two persons at risk), the World Health Organization (WHO) recommends delivering LLINs free-of-charge through mass distribution campaigns every 3 years [7]. Such mass campaigns have been shown to increase equitable ownership of LLINs [8], but achieving high LLIN coverage remains a challenge [9].

In 2021, Uganda ranked third in number of malaria cases globally [1]. Uganda’s Ministry of Health has committed to ensuring high LLIN coverage through mass distribution campaigns carried out every 3 years according to WHO guidelines. In Uganda, the first LLIN distribution campaign was conducted in 2013–14, delivering over 20 million LLINs to households free-of-charge nationwide [10]; a subsequent campaign was conducted in 2017–18. These campaigns successfully increased household ownership of at least one LLIN to over 90%, but in 2015, only 62% of households were adequately covered by one LLIN for every two residents [11]. In 2017–18, prior to the second mass campaign, adequate LLIN coverage had fallen to 18% [12], and in 2019, adequate coverage following the 2017–18 mass campaign was only 54% [13].

Uganda’s Ministry of Health and partners led the third mass LLIN distribution campaign in 2020–21, distributing about 28 million LLINs nationwide during the COVID-19 pandemic [14]. To evaluate the impact of this campaign on LLIN ownership, coverage, and use in Uganda, a cross-sectional survey was conducted soon after LLIN distribution in 12 districts.

## Methods

### Study design and setting

**Fig. 1:**
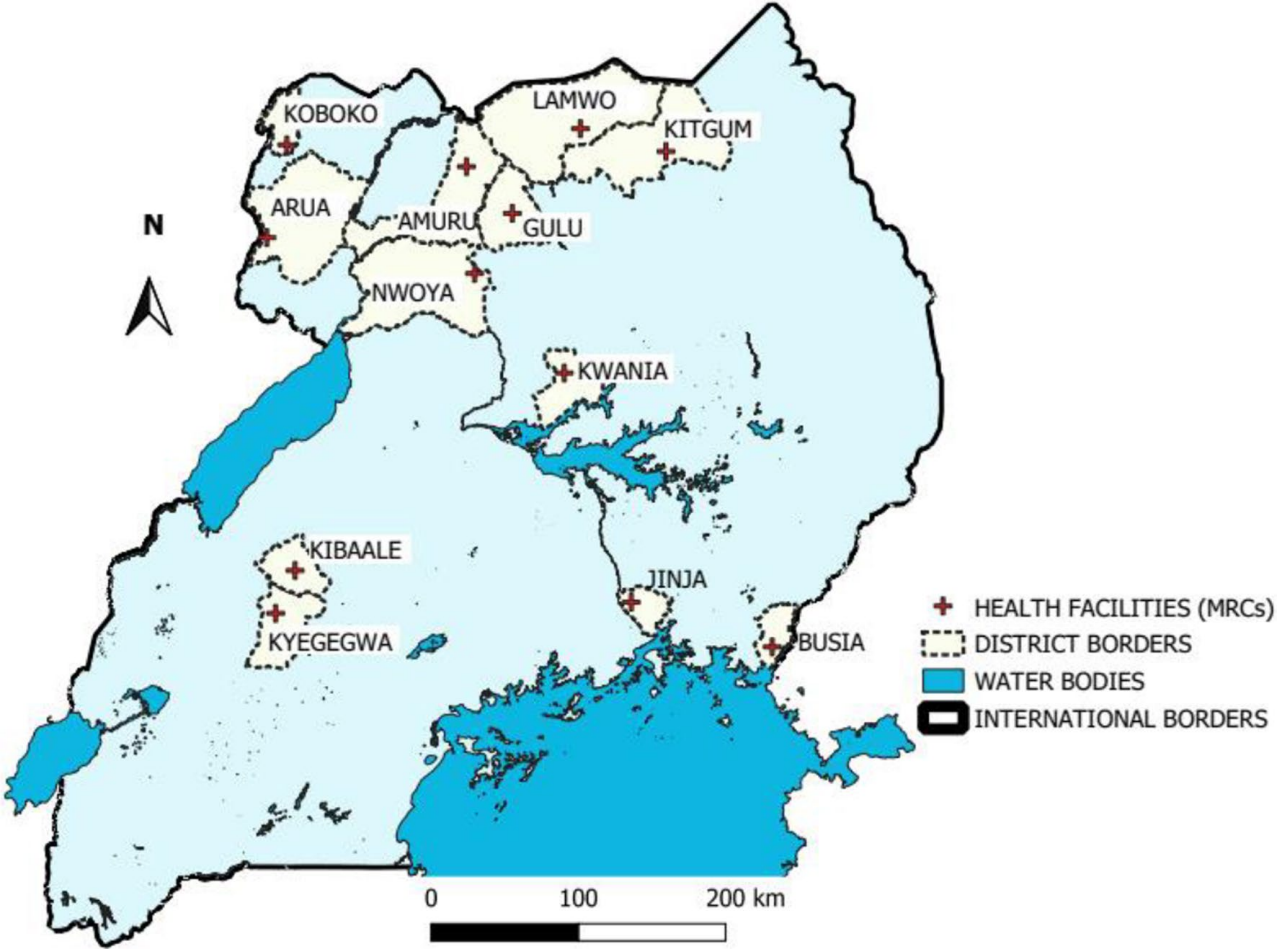
Map of Uganda showing the study districts, and public health facilities: Districts (white), public health facilities (bold red cross)

Target areas of 1–7 villages surrounding 12 government-run health facilities in 12 districts in Uganda were included (Fig. 1). These level III/IV health centres are located in moderate to high malaria transmission settings and have been established as Malaria Reference Centres (MRCs), sites for enhanced health facility-based malaria surveillance. These 12 MRCs were purposively selected to give good geographic spread of sites with some near borders with other countries, location in a rural area, and location along trade routes. The 12 sites were part of 64 MRCs included in a cluster-randomized trial to evaluate the impact of LLINs distributed in 2020–21 across 32 districts in Uganda (LLINEUP2, ClinicalTrials.gov: NCT04566510).

### Sample size and power determination

This survey was embedded in a cluster-randomized trial to evaluate the impact of LLINs distributed in 2020–21 across 32 districts in Uganda (ClinicalTrials.gov: NCT04566510). The sample size for the cross-sectional community surveys was predetermined for the main trial, using calculations applied for the outcome of parasite prevalence. A total of 50 households with at least one child aged 2–10 years were recruited from each cluster. A sample size of 634 households is estimated to provide a power of 100% for the primary outcome of adequate coverage with UCC LLINs, assuming baseline (pre-distribution) LLIN coverage of 17.9% and post-distribution coverage of 54.0% [12, 13], a level of significance of 0.05, and design effect of 2 [15].

### Sampling procedure, recruitment and enrollment

Target areas surrounding each MRC were identified, including the village where the MRC is located and adjacent villages that met the following criteria: (1) did not contain another government-run health facility, (2) located in the same sub-county as the MRC, and (3) similar incidence of malaria as the MRC’s village. All households within the MRC target areas were mapped and enumerated to generate a sampling frame for the survey. A random sample of enumerated households was selected from each target area to generate a list of households to approach for recruitment. Households on the recruitment list were approached and enrolled if the following criteria were met: (1) at least one adult aged 18 years or older present, (2) adult is a usual resident who slept in the sampled household on the night before the survey, and (3) agreement of the adult resident to provide informed consent. For each MRC target area, consecutive eligible households were surveyed until 50 households with at least one child aged 2–10 years were enrolled.

### Household registration and LLIN distribution

LLINs were distributed in the LLINEUP2 study sites by the Ugandan Ministry of Health through the national universal coverage campaign (UCC) in December 2020 (369,317 LLINs) and March 2021 (603,064 LLINs). LLIN distribution was conducted according to detailed national guidelines based on prior experiences from UCCs conducted in 2013–2014 and 2017–18 [16]. The guidelines were adapted to adhere to COVID-19 standard operating procedures. This campaign was conducted by multidisciplinary team who went door-to-door to register households and distribute LLINs. Data were managed through an Electronic Data Management Information System (EDMIS), which calculated the number of LLINs to allocate to each household, based on the registration data. The LLINs were issued to the head of household, or another adult resident. The target was to distribute one LLIN for every two household residents; with households of more than 10 residents receiving a maximum of 5 LLINs.

### Data collection and management

Household surveys were administered to heads of household or their designate using electronic questionnaires on hand-held tablet computers, which were programmed to include range checks and internal consistency checks. Information was gathered on characteristics of households and residents, proxy indicators of wealth including ownership of assets, and ownership and use of LLINs in the households. To objectively identify the LLINs, the RAs observed the nets within each household surveyed, and recorded the details of each net/LLIN. Data collected were transferred daily to a secure server on a private network at the core data facility in Kampala.

### Statistical analysis

Data were analysed using Stata version 14.1 (College Station, TX). Principal component analysis was used to generate a wealth index based on ownership of common household items. Households were ranked by wealth scores and grouped into terciles to provide a categorical measure of socioeconomic status. Modern houses were defined as having plaster or cement walls, metal or wooden roofs, and closed eaves; all other houses were defined as traditional [17]. The primary outcome was adequate UCC LLIN coverage (proportion of households that own at least one UCC LLIN for every two residents), and other outcome measures included: (1) UCC LLIN ownership (defined as the proportion of households that own at least one UCC LLIN), (2) LLIN access (proportion of residents who could sleep under UCC LLIN, if each UCC LLIN in the household were used by up to two residents), and (3) LLIN use (the proportion of household residents who slept under any LLIN the previous night).

Associations between variables of interest and outcome measures including adequate UCC LLIN coverage and LLIN use were estimated using multivariate logistic regression models with robust standard errors adjusted for clustering at the level of the health facility. Measures of association were expressed as odds ratios (ORs). A two-sided p-value of < 0.05 was considered statistically significant. Associations between variables of interest with ownership of UCC LLINs were also explored (Additional file 1).

## Results

### Characteristics of the households, and residents surveyed

**Fig. 2:**
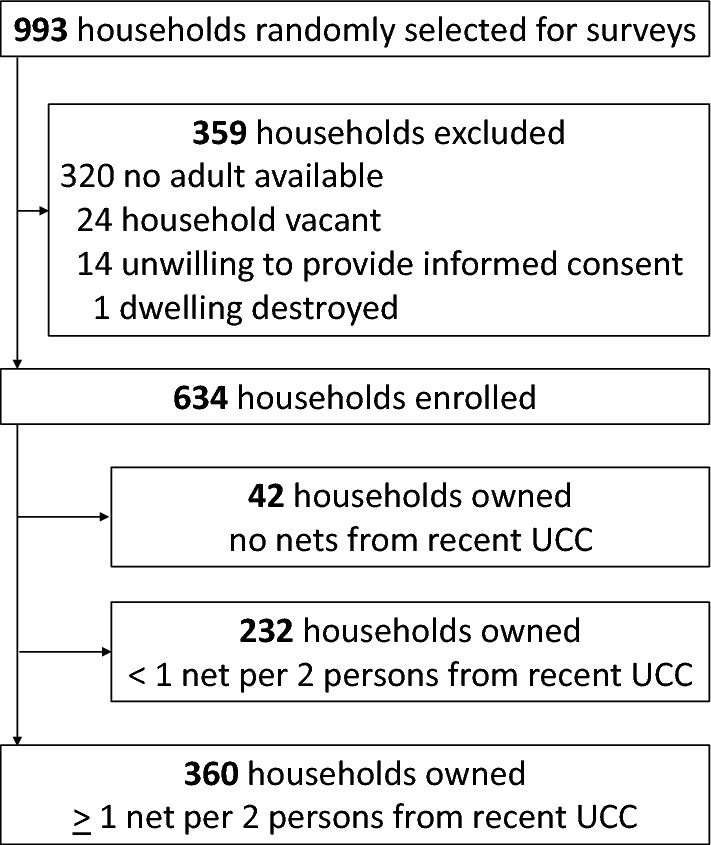
Study profile

Of 993 households approached for recruitment (Fig. 2), 359 were excluded before enrollment, primarily because no adult was available (89.1%). Most household heads were male (Table 1), with a median age of 40 years (range 18–95 years). The median number of household residents was 5 (range 1–14); 153 households (24.1%) had 7 or more residents. Most households had at least one child under 5 years, and were constructed with traditional materials. Close to a third of households were located ≥ 2 km from the nearest health facility.

**Table 1:** Characteristics of households and residents

Characteristic	Category	Findings n (%)
Household characteristics (N = 634)		
Gender of household head	Male	433 (68.3)
	Female	201 (31.7)
Age of the household head	median (range)	40 (18–95)
	18–39 years	303 (47.8)
	40–49 years	131 (20.7)
	50–85 years	200 (31.6)
Number of household residents	median (range)	5 (1–14)
	1–4	264 (41.6)
	5–6	217 (34.2)
	7–14	153 (24.1)
At least one resident < 5 years of age, n (%)		431 (68.0)
Distance to nearest health facility (km)	median (range)	1 (< 1–8)
	< 1 km	276 (43.5)
	1- < 2 km	175 (27.6)
	2 or more km	183 (28.9)
House type, n (%)	Traditional	461 (72.7)
	Modern	173 (27.3)
Socioeconomic index in terciles, n (%)	Poorest	212 (33.4)
	Poor	214 (33.8)
	Least poor	208 (32.8)
Characteristics of household residents (N = 3342)		
Gender	Male	1584 (47.4)
	Female	1758 (52.6)
Age in years	< 5 years	593 (17.7)
	5–15 years	1182 (35.4)
	> 15 years	1567 (46.9)
Relationship to head of household	Head of household	634 (19.0)
	1st degree	2220 (66.4)
	2nd degree/unrelated	478 (14.6)

Overall, 3342 household residents were surveyed (Table 1). About half of the residents were female, and over 15 years of age. Most residents were either the head of household (19.0%) or their first degree relative (66.4%); fewer residents were more distantly, or not, related to the household head (14.6%).

### Impact of the 2020–21 UCC on LLIN ownership and coverage

LLINs were distributed through the UCC to surveyed households in December 2020 (n = 475) and March 2021 (n = 159). Most households (86.4%) reported receiving some education about malaria and LLINs when the UCC nets were distributed. LLIN ownership was high in the households enrolled in the survey (Fig. 2, Table 2); 609 of 634 households (96.1%) owned at least one LLIN, 25 (3.9%) did not own a LLIN of any type, and 42 (6.6%) did not own a LLIN distributed through the UCC campaign. Of those households that did not own LLINs, most (59.5%) were away when nets were distributed, 19.1% reported that nets had run out, 16.6% were never told or did not collect the nets, and 4.8% households did not register or their local leaders refused to accept nets.

**Table 2:** LLIN indicators following Uganda’s universal coverage campaign (UCC) to deliver LLINs nationwide in 2020–2021

Characteristic	Category	Findings n (%)
Households (N = 634)		
Timing of LLIN distribution, n (%)	December 2020	475 (74.9)
	March 2021	159 (25.1)
Households that owned at least one UCC LLIN		592 (93.4)
Households that owned at least one LLIN (of any type)		609 (96.1)
Households with one UCC LLIN per two residents		360 (56.8)
Households with one LLIN (any type) per two residents		374 (59.0)
Households with any non-UCC LLIN		100 (15.8)
Households that received some education with the LLIN during the 2020–21 UCC campaign, n (%)		548 (86.4)
Residents (N = 3342)		
Residents who had access* to a UCC LLIN within their household		2642 (79.1)
Residents who had access* to any LLIN within their household		2744 (82.1)
Residents who slept under a UCC LLIN the prior night		2374 (71.0)
Residents who slept under any LLIN the prior night		2592 (77.6)
Bed nets (N = 1631)		
Any type of LLIN		1617 (99.1)
UCC LLIN (delivered 2020–2021)		1458 (89.4)
^£^UCC LLINs hung		1322 (90.7)
^£^UCC LLINs hung and used the previous night		1301 (89.2)

^*^Access defined as the proportion of residents who could sleep under an LLIN, if each LLIN in the household were used by up to two residents

^£^Denominator for these two indicators was the number of UCC LLINs (n = 1458)

**Fig. 3:**
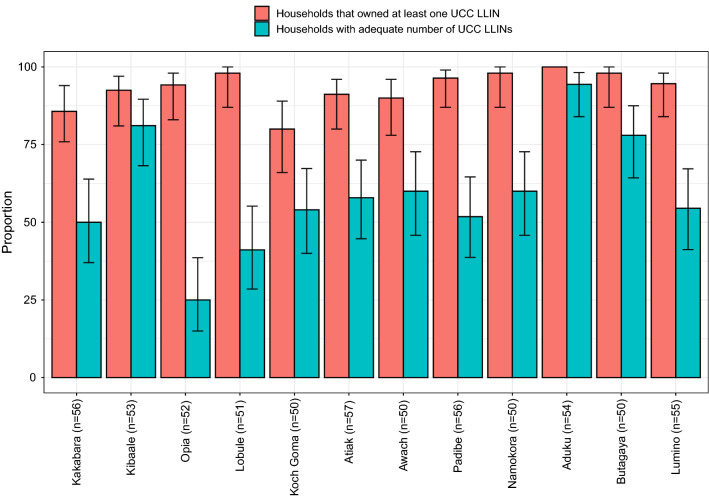
Proportions of owning at least one LLIN, and adequate LLINs among households across study sites

Despite high LLIN ownership, far fewer households were adequately covered by LLINs. Of the 634 households, 360 (56.8%) owned at least one UCC LLIN for every 2 residents, and 374 (59.0%) were adequately covered by LLINs of any type. Adequate coverage with UCC LLINs varied markedly by study site, from 32.7% to 94.4% in the different districts (Fig. 3).

### Impact of the 2020–21 UCC on LLIN use

Of the 3342 residents surveyed (Table 2), 79.1% had access to UCC LLINs (defined as the proportion of residents who could sleep under an LLIN, if every LLIN in the household was used by up to two residents); slightly more residents had access to an LLIN of any type. Most residents reported sleeping under a UCC LLIN the previous night (71.0%), and even more slept under any LLIN. Of the 1631 nets observed, nearly all were LLINs (99.1%), and most were distributed during the 2020–21 UCC (89.4%). Most UCC LLINs were hung, and had been used the previous night.

### Factors associated with adequate coverage by UCC LLINs

In an adjusted analysis, the factor most strongly associated with adequate coverage with UCC LLINs was fewer household residents (Table 3); only 29.7% of households with 7–14 residents were adequately covered with UCC LLINs vs 82.2% of those with 1–4 residents (adjusted odds ratios [aOR] 12.96, 95% CI 4.76–35.26, p < 0.001); vs 58.7% of those households with 5–6 residents (aOR 2.99, 95% CI 1.21–7.42, p < 0.018). Other factors associated with adequate coverage with UCC LLINs included household ownership of UCC nets only (64.3% vs 39.0% in households that owned non-UCC LLINs; aOR 3.55, 95% CIs 2.02–6.25, p < 0.001), older household heads (65.3% in households led by heads aged 50–85 years vs 57.2% in those led by heads aged 18–39 years; aOR 2.34, 95% CIs 1.54–3.56, p < 0.001), female heads of household (78.1% vs 52.8% in households led by males; aOR 1.87, 95% CIs 1.17–3.00, p = 0.009) and greater household wealth (63.8% in least poor vs 60.7% in poorest households, aOR 1.92, 95% CI 1.37–2.69, p < 0.001).

**Table 3:** Factors associated with a household receiving an adequate number of UCC LLINs (1 UCC LLIN per 2 residents)

Characteristic	Category	Outcome present n (%)	Univariate analysis*		Multivariate analysis*	
			OR (95% CI)	p-value	OR (95% CI)	p-value
Gender of the head of the household	Male	214 (52.8)	Reference	–	Reference	–
	Female	146 (78.1)	2.88 (1.87–4.44)	< 0.001	1.87 (1.17–3.00)	0.009
Age of the head of the household	18–39 years	155 (57.2)	Reference	–	Reference	–
	40–49 years	79 (61.7)	1.11 (0.58–2.13)	0.748	2.03 (0.91–4.51)	0.083
	50–85 years	126 (65.3)	1.45 (1.00–2.11)	0.051	2.34 (1.54–3.56)	< 0.001
Number of household residents	7–14	44 (29.7)	Reference	–	Reference	–
	5–6	122 (58.7)	2.53 (1.19–5.38)	0.016	2.99 (1.21–7.42)	0.018
	1–4	194 (82.2)	10.03 (3.73–26.97)	< 0.001	12.96 (4.76–35.26)	< 0.001
At least one resident < 5 years of age	Yes	230 (56.8)	Reference	–	Reference	–
	No	130 (69.5)	1.90 (1.22–2.96)	0.004	1.38 (0.90–2.12)	0.145
Timing of UCC distribution	December 2020	254 (57.1)	Reference	–	Reference	–
	March 2021	106 (72.1)	1.96 (0.70–5.48)	0.198	1.81 (0.57–5.72)	0.312
Socioeconomic index	Poorest	116 (60.7)	Reference	–	Reference	–
	Poor	117 (57.9)	0.85 (0.53–1.35)	0.485	1.32 (0.82–2.13)	0.246
	Least poor	127 (63.8)	0.88 (0.60–1.30)	0.516	1.92 (1.37–2.69)	< 0.001
House type	Modern	104 (66.2)	Reference	–	Reference	–
	Traditional	256 (58.9)	0.77 (0.54–1.10)	0.150	1.12 (0.68–1.86)	0.651
Distance to nearest health facility	< 1 km	167 (63.5)	Reference	–	Reference	–
	1- < 2 km	95 (57.9)	0.96 (0.73–1.25)	0.741	0.81 (0.56–1.16)	0.244
	2 or more km	98 (59.4)	1.18 (0.64–2.21)	0.593	1.41 (0.73–2.73)	0.304
Any non-UCC LLINs present after UCC	Yes	32 (39.0)	Reference	–	Reference	–
	No	328 (64.3)	2.98 (1.83–4.84)	< 0.001	3.55 (2.02–6.25)	< 0.001

^*^The analysis included only houses who received at least one UCC LLIN (N = 592), and was adjusted for clustering at the level of the MRC

### Factors associated with use of any LLIN by residents living in households with, and without, adequate UCC LLIN coverage

Of the 3166 residents living in households that owned at least one UCC LLIN, 1684 (53.2%) lived in households that were adequately covered by UCC LLINs, while 1482 (46.8%) lived in households that were inadequately covered (Table 4). The proportion of residents who reported sleeping under any LLIN the previous night was significantly higher in households that were adequately covered with UCC LLINs than in inadequately covered households (89.9% vs 69.8%, p < 0.001).

**Table 4:** Factors associated with LLIN use (slept under any LLIN the previous night) in residents living in households with and without adequate coverage of UCC LLINs (1 UCC LLIN per 2 residents)

Variable	Category	Household with adequate UCC LLINs n (%)	Multivariate analysis		Household without adequate UCC LLINs n (%)	Multivariate analysis	
			OR (95% CI)	p-value		OR (95% CI)	p-value
Gender of resident	Male	699 (88.4)	Reference	–	496 (69.6)	Reference	–
	Female	814 (91.2)	1.50 (1.00–2.25)	0.049	538 (70.0)	1.20 (0.83–1.74)	0.334
Age of resident	5–15 years	474 (85.1)	Reference	–	340 (59.5)	Reference	–
	< 5 years	294 (95.5)	2.52 (1.53–4.16)	< 0.001	202 (80.8)	3.04 (2.08–4.46)	< 0.001
	> 15 years	745 (91.0)	1.10 (0.59–2.05)	0.770	492 (74.4)	1.49 (0.98–2.27)	0.063
Timing of UCC distribution	March 2021	443 (86.5)	Reference	–	184 (61.5)	Reference	–
	December 2020	1070 (91.3)	1.66 (1.12–2.48)	0.013	850 (71.9)	1.69 (0.78–3.68)	0.187
Socioeconomic index	Least poor	546 (88.4)	Reference	–	329 (66.1)	Reference	–
	Poor	510 (90.4)	1.45 (0.90–2.34)	0.128	375 (69.2)	1.09 (0.69–1.73)	0.697
	Poorest	457 (91.0)	1.35 (0.77–2.39)	0.293	330 (74.7)	1.10 (0.56–2.17)	0.781
Distance to nearest health facility	2 or more km	411 (89.0)	Reference	–	291 (70.3)	Reference	–
	1- < 2 km	383 (86.5)	0.74 (0.33–1.68)	0.472	299 (69.4)	0.81 (0.51–1.30)	0.389
	< 1 km	719 (92.3)	1.64 (0.65–4.09)	0.292	444 (69.7)	0.82 (0.43–1.57)	0.548
Number of household residents	> = 7	299 (84.7)	Reference	–	587 (66.9)	Reference	–
	5–6	605 (90.2)	1.65 (0.99–2.77)	0.055	348 (76.3)	1.47 (1.05–2.06)	0.026
	< = 4	609 (92.3)	2.19 (0.96–5.00)	0.063	99 (66.9)	0.85 (0.59–1.22)	0.378
Household with at least one resident < 5 years of age	No	444 (84.1)	Reference	–	234 (64.5)	Reference	–
	Yes	1069 (92.5)	2.52 (1.62–3.93)	< 0.001	800 (71.5)	1.11 (0.76–1.60)	0.597
Relationship to head of household	2nd degree/unrelated	223 (87.5)	Reference	–	130 (59.4)	Reference	–
	1st degree	947 (88.6)	1.13 (0.76–1.68)	0.554	710 (68.9)	1.58 (1.02–2.45)	0.039
	Head of HH	343 (95.3)	3.29 (1.30–7.72)	0.006	194 (83.6)	3.94 (2.38–6.51)	< 0.001
Any non-UCC LLINs present after UCC	No	1369 (89.4)	Reference	–	791 (68.8)	Reference	
	Yes	144 (94.1)	2.22 (1.19 (4.15)	0.012	243 (73.2)	1.67 (1.21–2.29)	0.002

Analysis included only household residents in houses who received at least one UCC LLIN (N = 3166; those with adequate UCC LLINs (n = 1684), and inadequate UCC LLINs (n = 1482)), and adjusted for clustering at the level of the MRC

In an adjusted analysis restricted to residents of households that were adequately covered by UCC LLINs (n = 1684), LLIN use was higher in children < 5 years (95.5% vs 85.1% in children aged 5–15 years; aOR 2.52, 95% CI 1.53–4.16, p < 0.001), in households with at least one child under 5 years (92.5% vs 84.1% in households without young children, aOR 2.52, 95% CI 1.62–3.93, p < 0.001), and in heads of household (95.3% vs 87.5% in residents who were more distantly (or not) related to the household head, aOR 3.29, 95% CI 1.30–7.72, p = 0.006). Other factors associated with LLIN use the previous night in households that were adequately covered by UCC LLINs included ownership of non-UCC LLINs, timing of the UCC LLIN distribution, and female gender.

In a similar adjusted analysis restricted to residents of households that were not adequately covered by UCC LLINs (n = 1482), LLIN use was higher in heads of household (83.6% vs 59.4% in residents who were more distantly related; aOR 3.94, 95% CI 2.38–6.51, p < 0.001), and in children < 5 years (80.8% vs 59.5% in children aged 5–15 years; aOR 3.04, 95% CI 2.08–4.46, p < 0.001). Other factors associated with use of any LLIN in households that were inadequately covered by UCC LLINs included household size and presence of non-UCC LLINs.

## Discussion

LLINs are the mainstay of malaria control in Uganda. To ensure Ugandans have access to LLINs, the Ministry of Health delivers free nets through mass distribution campaigns every 3–4 years. However, achieving and sustaining high LLIN coverage remains a challenge. To better understand the impact of Uganda’s campaign to distribute LLINs in 2020–21, and factors associated with LLIN coverage and use, a cross-sectional survey was conducted 1–5 months post-distribution in 12 districts across Uganda. Considering several key LLIN indicators, the 2020–21 campaign was a success. Over 93% of households owned at least one LLIN distributed through the 2020–21 UCC, and over 70% of residents reported sleeping under a UCC LLIN the previous night. However, less than 60% of households owned at least one UCC LLIN for every two residents, which varied substantially by site, suggesting that the number of nets distributed to many households was insufficient to ensure adequate coverage. Prior studies in Uganda have highlighted that net attrition is also a major problem [2, 12], with the lifespan of many LLINs less than the anticipated 3 years. Strategies to ensure that households receive enough nets to guarantee high coverage and access to LLINs must be employed in future mass distribution campaigns.

The WHO recommends distributing LLINs every 3 years through mass campaigns supplemented by delivery of LLINs through routine channels, such as antenatal clinics [7]. Uganda’s Ministry of Health and partners have maintained commitment to mass distribution campaigns, and the success of the 2020–21 campaign, despite the ongoing COVID-19 pandemic, is remarkable. Contributors to this success include the door-to-door distribution model, which allowed the team to access individual households. Lockdowns and movement restrictions imposed to mitigate the spread of SARS-CoV-2 in Uganda may have also confined adults at home, making it easier to locate them and to distribute LLINs to individual households. Use of an electronic database to register households and residents, and to allocate the number of LLINs at the household level, was another major advance; in prior UCCs registration data were entered by hand and aggregated to determine the number of LLINs to allocate at the level of the subcounty [18]. This aggregated approach was susceptible to errors of omission, leaving some households without LLINs. Future campaigns should leverage and build on the electronic database established for this campaign to more accurately estimate the number of LLINs needed in advance to guide procurement, and to calculate the number of LLINs required in each household. Although few households did not own a UCC LLIN, engaging with community members to ensure they are aware of the distribution plans, and are available when LLINs are delivered, is essential as LLIN ownership is the foundation to improve LLIN access, coverage, and use.

In this study, the strongest predictor of adequate coverage with UCC LLINs was the number of household residents; the odds of adequate coverage with UCC LLINs were 13 times higher in households with only 1–4 residents than in those with 7–14 residents. Wealthier households, and those led by older individuals and female heads of household, were also more likely to be adequately covered by UCC LLINs. In the 2020–21 campaign, the number of LLINs distributed to each household was restricted to a maximum of five nets, regardless of household size. The practice of capping the number of LLINs distributed was likely the major contributing factor to inadequate LLIN coverage [7]. In future LLIN campaigns, strategies to increase the number of nets available for distribution (starting with adequate procurement), and to ensure that large households receive the correct number of LLINs to cover all residents (avoiding any blanket approaches to restricting the number of LLINs distributed) should be adopted. Routine distribution channels should also be strengthened to fill any gaps resulting from insufficient delivery of nets in the mass campaigns, which will be compounded by net attrition over time. In Uganda, LLINs are routinely distributed to vulnerable groups through the Expanded Program on Immunization, antenatal clinics, schools, and community health workers. Approaches to expand these routine channels, and to target poorer households and households led by younger males, should be explored.

Achieving adequate LLIN coverage is an important step toward maximizing access and use of LLINs. In this study, 86% of residents of households that were adequately covered with UCC LLINs slept under a LLIN the previous night, compared to only 62% in households that were inadequately covered. This link between adequate LLIN coverage and use of LLINs has been demonstrated elsewhere [19–22]. In western Kenya, households with more residents were less likely to own adequate numbers of LLINs, which strongly reduced the likelihood of using LLINs [19]. Similarly, elsewhere in Africa, including the Democratic Republic of Congo, Zambia, and Madagascar, as well as in Papua New Guinea and India, LLIN use was significantly higher in households that were adequately covered, with low LLIN coverage the main barrier to LLIN use [20–24]. In Tanzania, 2 years following a mass campaign, larger households, including those with more than four residents, were less likely to have access to, and to use, LLINs [25]. In Ethiopia and southern Africa, a study of over 6,000 households found that household size was associated with lack of equality in LLIN ownership and use of LLINs [26, 27]. These findings suggest that improving LLIN coverage would likely increase use of LLINs.

School-aged children were less likely to use LLINs in this study than younger children or older residents, regardless of whether they lived in a household that was adequately covered with UCC LLINs, or not. LLIN use among children aged 5–15 years from inadequately covered households was particularly low (< 60%). Multiple studies have found that school-aged children are less well-covered by LLINs [10, 12, 28]. These older children are often overlooked by malaria control strategies, which have traditionally focused on children under-five and pregnant women who typically bear the burden of malaria morbidity and mortality in higher transmission areas [29, 30]. Older children, who have developed anti-disease immunity through repeated exposure, are often asymptomatic when infected with malaria parasites. As a result, malaria infections in this age group may be missed. Malaria in school-aged children is not benign, however; older children may suffer consequences of malaria including clinical malaria episodes, anaemia, and cognitive impairment [28, 31]. School-aged children typically have the highest prevalence of asymptomatic malaria infection in higher transmission areas, serving as important reservoirs of infection for onward transmission of malaria [32–34]. Moreover, as the epidemiology of malaria in Africa evolves in response to intensified control efforts, increasing urbanisation, and climate change, transmission intensity and exposure to malaria parasites will decline, and consequently, acquisition of immunity may be slower. As a result, older children could have less robust immunity to malaria, and be at higher risk of clinical consequences of malaria infection. Studies conducted in Uganda, and Mali report shifts in the burden of malaria to older children [35–37], supporting this theory. Strategies such as school-based interventions aiming to improve LLIN coverage and use in older children are gaining traction. In Tanzania, a study to evaluate a school-based LLIN distribution programme found that LLIN use increased from 57 to 77% among primary school children [38]. Targeting school-aged children, who can serve as agents of change, can also improve LLIN use within households [39].

In this study, relationship of household residents to the head of household was associated with LLIN use in all households, regardless of LLIN coverage, suggesting that hierarchy and status within households may influence LLIN access and use. The odds of using a LLIN were 3–4 times greater for heads of household compared to second-degree relatives or unrelated residents. In households that were inadequately covered by UCC LLINs, less than 60% of distantly related household residents used an LLIN. This finding is somewhat unexpected given that settlements in most African countries tend to function as close units, irrespective of whether household members are nuclear or extended family [40]. However, a prior survey in Uganda also identified relationship to the head of household as a factor strongly associated with LLIN use, with second-degree relatives and unrelated household members less likely to use LLINs that household heads, regardless of adequate LLIN coverage [12]. Similarly, in Kenya, non-nuclear family members were less likely to use nets than residents closely related to the head of household (nuclear vs nonnuclear members, aOR = 4.75 (2.89–7.81) and aOR = 4.16 (1.40–12.38) in highland areas and lowlands respectively) [19]. In this study 15% of household residents were second-degree relatives or not related to the head of household. However, we were not able to explore these relationships, or to gain an understanding of whether these residents are permanent, or more transient, members of the household. An in-depth understanding of this population, including their role in the household and household dynamics is needed, to ensure that this vulnerable group is better targeted in future LLINs campaigns and interventions.

This study had several limitations. First, an in-depth understanding of why household residents use LLIN, or not, is limited by the quantitative nature of the questionnaire used in this survey. Further exploration of these important issues using qualitative research methods is needed to better understand determinants of LLIN use within households. Second, target areas included only a few villages surrounding the MRCs, which may have limited generalizability of these findings. However, all statistical models adjusted for site-specific clustering, to minimize any bias arising from the non-representativeness of few villages, and included conservative standard errors [41]. Third, LLIN use was self-reported, which could have under- or over-estimated actual LLIN use, but is the standard approach to measuring LLIN use [42].

## Conclusion

The 2020–21 mass campaign to distribute LLINs in Uganda was a success, as evidenced by high ownership of UCC LLINs soon after distribution. However, far fewer households were adequately covered by UCC LLINs (one UCC LLIN for every two residents), specifically larger households with 7 or more residents. Children aged 5–15 years, and residents who were distantly related to the head of household, were less likely to use LLINs, particularly in households that were inadequately covered by UCC LLINs. In future LLIN campaigns, strategies to increase the number of nets available for distribution, and to ensure that large households receive the correct number of LLINs to cover all residents, should be adopted. Understanding the complexities of contextual and behavioural drivers of LLIN use is needed to guide future interventions, educational messages, and behavioural change communication strategies. Malaria control programmes should re-focus on school-aged children, who contribute disproportionately to malaria transmission [43], and may be at greater risk of clinical consequences of malaria as the epidemiology of malaria in Africa changes.

## Supplementary Information

**Additional file 1:** **Table S1**. Factors associated with households owning at least one UCC LLIN

## Data Availability

The datasets reported herein will be made publicly available on completion of the LLINEUP2 project but are available from the corresponding author on reasonable request.

## Abbreviations

AL: Artemether-lumefantrine
ACTs: Artemisinin-based combination therapies
DHS: Demographic health survey
EDMIS: Electronic data management information system
IDRC: Infectious disease research collaboration
IRS: Indoor residual spraying
LLIN: Long lasting insecticidal net
MIS: Malaria indicator survey
MOH: Ministry of health
MOLAB: Molecular research laboratory
MRC: Malaria reference centre
OR: Odds ratio
PCR: Polymerase chain reaction
PBO: Piperonyl butoxide
RDT: Rapid diagnostic test
UCC: Universal coverage campaign
WHO: World Health Organization
