# Supplementary material for: LLIN Evaluation in Uganda Project (LLINEUP2)—Factors associated with coverage and use of long‑lasting insecticidal nets following the 2020–21 national mass distribution campaign: a cross-sectional survey of 12 districts
Source: Malar J. 2022 Oct 19;21:293. doi: 10.1186/s12936-022-04302-7 (PMC9580445; doi:10.1186/s12936-022-04302-7)
Supplement: Supplementary file 1 — Additional file 1: Table S1. Factors associated with households owning at least one UCC LLIN [file 12936_2022_4302_MOESM1_ESM.docx]

Supplementary file

| **Characteristic** | **Category** | **Outcome present n (%)** | **Univariate analysis*** | | **Multivariate analysis*** | |
| --- | --- | --- | --- | --- | --- | --- |
|  |  |  | **OR (95% CI)** | **p-value** | **OR (95% CI)** | **p-value** |
| Gender of the head of the household | Male | 405 (93.5) | Reference | - | Reference | - |
|  | Female | 187 (93.0) | 1.07 (0.56-2.07) | 0.832 | 1.48 (0.66-3.34) | 0.346 |
| Age of the head of the household | 18-29 years | 100 (84.0) | Reference | - | Reference | - |
|  | 30-49 years | 299 (94.9) | 3.65 (1.45-9.19) | 0.006 | 3.15 (0.96-10.31) | 0.058 |
|  | 50-85 years | 193 (96.5) | 4.62 (1.64-13.03) | 0.004 | 5.19 (1.90-14.19) | 0.001 |
| Number of household residents | 1-4 | 236 (89.4) | Reference | - | Reference | - |
|  | 5-6 | 208 (95.9) | 2.96 (1.31-6.70) | 0.009 | 2.50 (1.22-5.11) | 0.012 |
|  | 7-14 | 148 (96.7) | 3.09 (1.15-8.26) | 0.025 | 2.16 (0.75-6.24) | 0.155 |
| At least one resident < 5 years of age | No | 187 (92.1) | Reference | - | Reference | - |
|  | Yes | 405 (94.0) | 1.30 (0.56-3.01) | 0.540 | 1.72 (0.67-4.44)) | 0.264 |
| Timing of UCC distribution | March 2021 | 147 (92.5) | Reference | - | Reference | - |
|  | December 2020 | 445 (93.7) | 1.26 (0.44-3.58) | 0.666 | 1.23 (0.54-2.78) | 0.623 |
| Socioeconomic index | Poorest | 191 (90.1) | Reference | - | Reference | - |
|  | Poor | 202 (94.4) | 1.73 (0.98-3.07) | 0.060 | 2.21 (0.79-6.19) | 0.130 |
|  | Least poor | 199 (95.7) | 2.28 (1.30-3.99) | 0.004 | 3.24 (1.15-9.12) | 0.026 |
| House type | Modern | 157 (90.8) | Reference | - | Reference | - |
|  | Traditional | 435 (94.4) | 1.85 (0.97-3.53) | 0.061 | 3.04 (1.24-7.47) | 0.015 |
| Distance to nearest health facility | 2 or more km | 165 (90.2) | Reference | - | Reference | - |
|  | 1-<2 km | 164 (93.7) | 1.21 (0.60-2.41) | 0.597 | 1.01 (0.63-1.60) | 0.982 |
|  | < 1 km | 263 (95.3) | 1.61 (0.70-3.68) | 0.259 | 1.52 (0.59-3.93) | 0.388 |
| Any non-UCC LLINs present after UCC | Yes | 82 (82.0) | Reference | - | Reference | - |
|  | No | 510 (95.5) | 6.89 (4.14-11.46) | <0.001 | 10.85 (5.83-20.19) | <0.001 |

**Table S1. Factors associated with households owning at least one UCC LLIN**

*adjusted for clustering at the level of the MRC
